# Cytotoxic T-cell activation profile in critically ill children with malignancies and hemophagocytic lymphohistiocytosis

**DOI:** 10.1038/s41390-025-03962-w

**Published:** 2025-03-05

**Authors:** Sijuan Sun, Yue Liu, Hui Zhao, Yan Miu, Xiaohang Huang, Shuhong Shen, Hong Ren, Jian Zhang

**Affiliations:** 1 Department of Pediatric Intensive Care Unit, Shanghai Children’s Medical Center, Shanghai Jiao Tong University School of Medicine, Shanghai, China; 2 Department of Pediatrics, Shanghai Jiading District Anting Hospital, Shanghai, China; 3 Department of Hematology/Oncology, Shanghai Children’s Medical Center, Shanghai Jiaotong University School of Medicine, Shanghai, China

## Abstract

**Background:**

The early identification of hemophagocytic lymphohistiocytosis (HLH) in critically ill children with malignancies is challenging. The value of an activated cytotoxic T-cell profile in diagnosing HLH in this group of patients is unknown.

**Methods:**

Critically ill children with malignancies who suffered from persistent cytopenia in the pediatric intensive care unit were included. Children were divided into two groups based on how many clinical HLH diagnostic criteria they fulfilled: M-HLH group, ≥5 criteria; hematologic malignancy (HM) group, ≤4 criteria. Flow cytometry tests were performed within 24 h after the patient’s admission.

**Results:**

Thirty-seven children who fulfilled the requirements were enrolled. Twenty children were classified into the M-HLH group and 17 into the HM group. The M-HLH group exhibited a higher mortality rate than the HM group. CD38 + HLA-DR + CD8+ T cells% and interferon-gamma (IFN-γ) were elevated in the M-HLH group. The area under the curve values of the two indexes were 0.906 and 0.897 respectively for the identification of M-HLH in the critically ill children, with CD38+/HLA-DR + CD8+ T cells% > 39.66% and IFN-γ > 22.58 exhibiting the best performance.

**Conclusion:**

Cytotoxic T-cell activation profile with CD38 + HLA-DR + CD8+ T cells% and IFN-γ is valuable in the early diagnosis of HLH in critically ill children with malignancies.

**Impact:**

- The early diagnosis of hemophagocytic lymphohistiocytosis in critically ill children with malignancies (M-HLH) remains a major challenge for intensivists.
- Cytotoxic T-cell activation profile with the frequency of CD38 + HLA-DR+ T cells in CD8+ T cells (CD38 + HLA-DR + CD8+ T cells%) and interferon-gamma (IFN-γ) is valuable in the early identification of pediatric M-HLH.
- These findings will support the future implementation of T-cell activation markers in the clinical management of children with M-HLH.

## Introduction

Hemophagocytic lymphohistiocytosis (HLH) is a rapidly progressive and highly fatal disease with a mortality rate of more than 30% in pediatric patients.^1,2^ According to Histiocyte Society’s HLH-2004 criteria, a clinical diagnosis of HLH can be made by fulfilling five out of eight clinical features, including fever, cytopenia, splenomegaly, hypertriglyceridemia or hypofibrinogenemia, hemophagocytosis, NK cell dysfunction, and elevated levels of ferritin and soluble IL-2 receptor (sIL-2R).^3^ Because of immune dysregulation associated with primary malignancy, malignancy-associated treatment, or comorbid severe infection, HLH-like symptoms are not uncommon in critically ill children with malignancies, making their early diagnosis of malignancy-associated HLH (M-HLH) quite challenging, and the estimated 1-year overall survival rate of adults and children is less than 40%.^4,5^ Searching for early sensitive and specific biomarkers in the diagnosis of HLH is imperative for critically ill children with malignancies.

The pathophysiology of HLH is based on the persistent activation of T cells and macrophages, inducing severe inflammatory response with multiple organ dysfunction and even death, and uncontrolled T-cell activation and secreted interferon-gamma (IFN-γ) are the key drivers of HLH.^6^ In routine laboratory tests, high levels of sIL-2R and ferritin have been used for the diagnosis of M-HLH.^7^ Nevertheless, both indexes lack specificity in patients, limiting their use in the context of early intervention.

CD38 is a type II transmembrane immunomodulatory protein expressed on thymocytes and different immune cells.^8^ Co-expression of CD38 and HLA-DR on CD8 + T cells is the hallmark of cytotoxic T-cell activation, which expresses genes involved in cell proliferation, glycolysis, and cytotoxicity; tends to polarize Tc1 cells; and secretes IFN-γ and tumor necrosis factor in response to external stimuli.^9,10^ Recently, the frequency of CD38 + HLA-DR + T cells in CD8 + T cells (CD38 + HLA-DR + CD8 + T cells%) was reported to exhibit potential application value in the differentiation of HLH from early sepsis in children,^9^ however, its early diagnostic value to identify the HLH in a relatively large cohort of critical children with malignacies is still unknown.

This study aims to present the value of cytotoxic T-cell activation profile (CD38 + HLA-DR + CD8 + T cells% and IFN-γ) in the early diagnosis of M-HLH in critically ill children.

## Methods

### Population

**Fig. 1:**
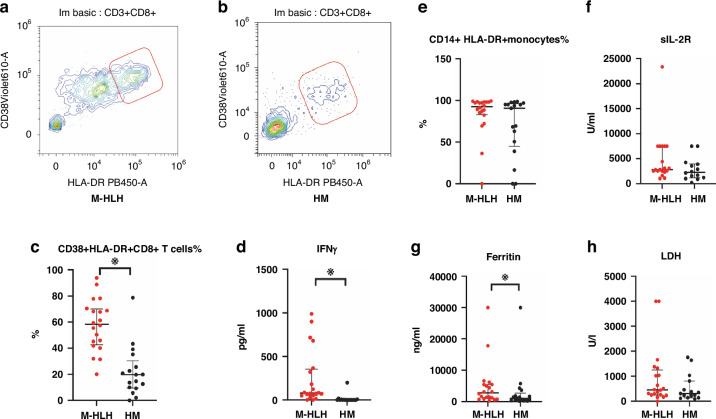
Peripheral blood cell activation profile and inflammation indexes. **a** Representative Flow cytometry plots of CD38 + HLA-DR + CD8+ T cells in M-HLH (**a**) and HM (**b**) groups. **c**–**h** Comparison of CD38 + HLA-DR + CD8+ T cells%, IFNγ, CD14^+^HLA-DR^+^ monocyte%, sIL-2R, Ferritin, and LDH in patients of the two groups. LDH lactate dehyrogenase. Independent-samples Mann–Whitney *U* test was used for the comparison of two groups, Bars represent the median and interquartile range, ※indicates *p* < 0.05.

This retrospective observational study in a tertiary children’s medical center was approved by the Institutional Review Board (No. SCMCIRB-K2024117-1). From December 2022 to July 2024, critically ill children with malignancies who were transferred to the pediatric intensive care unit and suffered from persistent two- or three-line cytopenia (persistent for at least 3 days) were enrolled. The patients were divided into two groups based on how many HLH-2004 criteria they fulfilled: M-HLH group, ≥5 criteria; hematologic malignancy (HM) group, ≤4 criteria. In the absence of an NK activity test, more than six criteria were required to be fulfilled. Children treated with chimeric antigen receptor T-cell immunotherapy or hematopoietic stem cell transplantation were excluded. Cell activation indexes such as CD38 + HLA-DR + CD8+ T cells% (gated on CD8+ T cells; Fig. 1a, b), CD14 + HLA-DR+ monocytes% (gated on CD14+ monocytes), and IFN-γ were tested by flow cytometry using peripheral blood samples within 24 h of patient’s admission. The Pediatric Sequential Organ Failure Assessment (pSOFA) and the Pediatric Risk of Mortality (PRISM) III score were used for clinical severity evaluation.^11,12^ Sepsis was diagnosed using the following criterion: infection with pSOFA score ≥ 2.

### Statistical analysis

IBM SPSS Statistics (Version 27) and GraphPad Prism 10 (Version 10.2.0) were used for data analysis or graph plotting. Independent-samples Mann–Whitney *U* test was used to compare the differences in indexes between groups depending on whether the data conform to a Gaussian distribution. Receiver operating characteristic (ROC) analysis was used to test the efficacy of cytotoxic T-cell activation indexes to differentiate pediatric M-HLH from HM. Pearson’s correlation was used to detect the correlation between cytotoxic T-cell activation indexes and clinical severity scores.

## Results

### General characteristics of patients

A total of 64 critically ill children with malignancies and cytopenia were enrolled, of which nine patients underwent chimeric antigen receptor T-cell immunotherapy therapy, 10 patients had hematopoietic stem cell transplantation, and eight children who did not complete more than six tests of HLH diagnostic criteria were excluded. The data of 37 patients were finally used for analysis. Twenty children were classified into the M-HLH group, with primary diseases including 10 cases of leukemia, 4 cases of lymphoma, and 6 cases of post-transplantation lymphoproliferative disorder. Seventeen children were classified into the HM group, with primary diseases including 14 cases of leukemia, 2 cases of lymphoma, and 1 case of idiopathic thrombocytopenic purpura. Bone marrow examinations were conducted in 18 patients from the M-HLH group and 15 patients from the HM group, with hemophagocytosis confirmed in only 3 cases within the M-HLH group. There was no statistically significant difference between the age and weight of the two groups (Table 1).

**Table 1:** General characteristics of patients

	M-HLH	HM
*N*	20	17
Gender (M/F)	12/8	14/3
Age (year)	7.09 ± 5.38	9.03 ± 3.83
weight (kg)	24.63 ± 15.32	30.66 ± 14.04
Primary Disease		
ALL	9	12
AML	1	2
Lymphoma	4	2
PTLD	6	0
ITP	0	1
*Sepsis	15/20 (75%)	5/17 (29.4%)
Bacteria	11/15 (73.3%)	4/5 (80%)
Viruses	7/15 (46.7%)	2/5 (40%)
Fungi	1/15 (6.7%)	1/5 (20%)
Cell activation files		
*CD38 + HLA-DR + CD8 + T cells % median (IQR)	58.39 (42.7–70.0)	19.74 (9.6–30.4)
*INFγ median (IQR) (pg/ml)	77.32 (51.6–355)	2.44 (2.44–5.4)
CD14 + HLA-DR+monocytes%	92.53 (83.07–98.11)	90.63 (44.75–96.03)
LDH median (IQR) (U/L)	452 (249–1252)	301 (141–807)
*Ferritin median (IQR) (ng/ml)	2757 (897–5381)	941 (354–2670)
sIL-2R median (IQR) (U/ml)	2777 (2500–7500)	2260 (1161–3950)
*pSOFA median (IQR)	12 (7.5–14)	8 (4–10)
PRISM III median (IQR)	15 (9–17.8)	12 (8.5–17)
Corticosteroid Use	20/20 100%	13/17 76.5%
High dose	40%	0%
Low dose	60%	100%
None	0	23.6%
Death	7/20 (35%)	2/17 (12%)

*ALL* Acute lymphoblastic leukemia, *AML* Acute myeloid leukemia, *ITP* idiopathic thrombocytopenic purpura, *PTLD* Post-transplant lymphoproliferative disorders, *represent *p* < 0.05.

The percentages of comorbid sepsis in the two groups were 75% (M-HLH) and 29.4% (HM). In the M-HLH group, 73.3% were infected with bacteria, and 46.7% had viral infections, mainly Epstein-Barr Virus (EBV) (5/7, 71.4%). Approximately 60% of the patients were treated with low-dose dexamethasone (0.1–0.5 mg/kg) or methylprednisolone (1–2 mg/kg) per day and 40% with high-dose glucocorticoids (methylprednisolone 10–20 mg/kg per day) to control the excessive inflammatory response within the first 48 h (Table 1).

### Peripheral blood cell activity profile and inflammation indexes

The values of CD38 + HLA-DR + CD8+ T cells%, IFN-γ, and ferritin were higher in the M-HLH group than in the HM group. Neither CD14 + HLA-DR+ monocytes% nor sIL-2R or lactate dehyrogenase (LDH) exhibited statistically significant differences among children between the two groups. The median values of CD14 + HLA-DR+ monocytes% in the M-HLH and HM groups were 92.53% and 90.63%, respectively (Table 1, Fig. 1c–h).

**Fig. 2:**
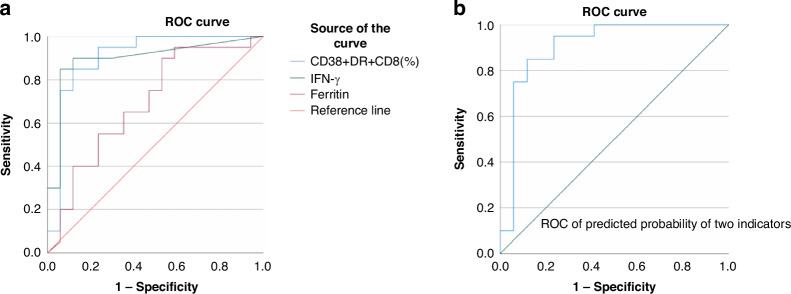
The ROC of T-cell activation profile and Ferritin in distinguishing cases of M-HLH from HM. **a** ROC curves of CD38 + HLA-DR + CD8+ T cells as a percentage of CD8+ T cells (AUC = 0.906, *p* < 0.01), IFNγ (AUC = 0.897, *p* < 0.01) and Ferritin (AUC = 0.693, *p* = 0.046). **b** ROC curve of the combined parameter: Predicted probability calculated by binary logistic regression with CD38 + HLA-DR + CD8+ T cells (%) and IFNγ (AUC = 0.906, *p* < 0.01).

ROC analysis indicated that the area under the curve values (AUC) of CD38+HLA-DR + CD8+ T cells%, IFN-γ, and ferritin were 0.906, 0.897, and 0.693, respectively (Fig. 2); AUC of the combined parameter: predicted probability calculated by binary logistic regression with CD38 + HLA-DR + CD8+ T cells (%) and INFγ was 0.906 (*p* < 0.01). The sensitivity and specificity of CD38+/HLA-DR + CD8+T cells% > 39.66% to differentiate critically ill children with M-HLH from those with HM were 85.0% and 88.2%, respectively; the sensitivity and specificity of IFN-γ > 22.58 to differentiate the two groups of children were 85% and 94%, respectively.

### Severity scores and prognosis

**Fig. 3:**
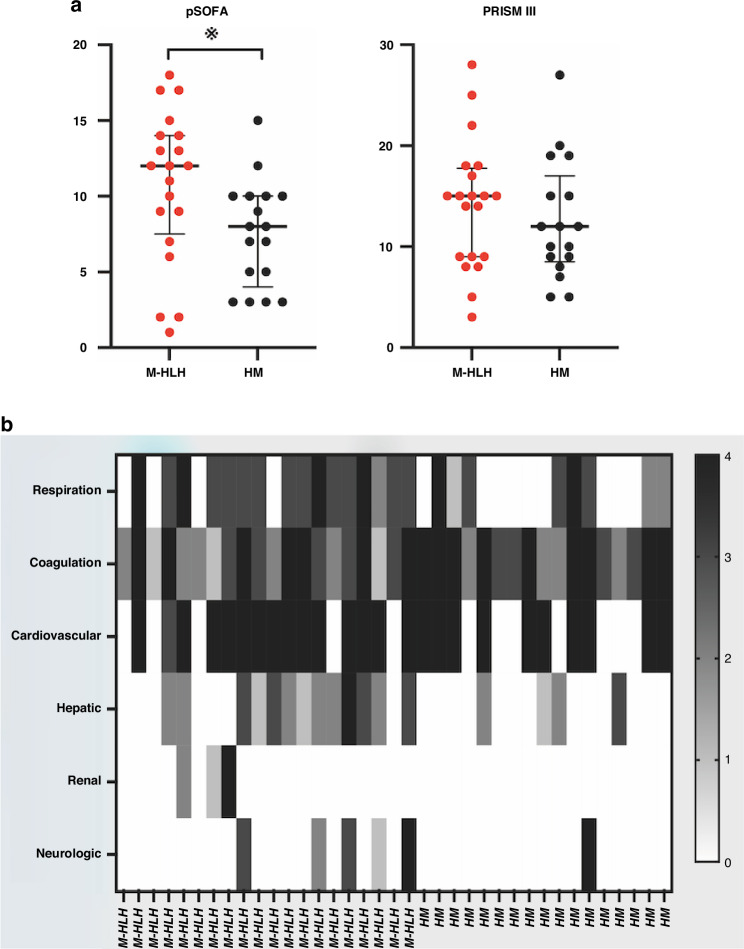
The clinical severity parameters and details of multiple organ dysfunction in patients. **a** Comparison of pSOFA and PRISM III score in patients of the two groups. Bars represent the median and interquartile range. **b** Heatmap of pSOFA score details of the patients. Gray scale of the bar represents the pSOFA score value. pSOFA Pediatric Sequential Organ Failure Assessment. PRISM Pediatric Risk of Mortality.

Children in the M-HLH group demonstrated significantly higher pSOFA score compared to those in the HM group, however, the Pediatric Risk of Mortality III (PRISM III) score did not differ significantly between the two groups. (Fig. 3a, Table 1). Further analysis of pSOFA evaluation indicated that in the M-HLH group, the most frequently involved organ systems were the cardiovascular, coagulation, and respiratory systems (Fig. 3b). There were seven deaths in the M-HLH group and two deaths in the HM group.

No statistically significant correlation was observed between severity scores and cytotoxic T-cell activation indices in pediatric patients within the M-HLH group (Table 2).

**Table 2:** Correlation of Cytotoxic T-cell activation indexes with severity scores in M-HLH group

	pSOFA	PRISM III
CD38+HLA-DR + CD8+ T cells%	*p* = 0.927	*p* = 0.786
IFNγ	*p* = 0.568	*p* = 0.730

## Discussion

Early intervention in the hyperinflammatory state is critical for reversing multiple organ failures and reducing mortality in children with malignancy-associated HLH. This approach not only helps to lower ICU costs but also, more importantly, enhances opportunities for subsequent treatment of the underlying disease. However, effective intervention relies on early identification, which remains a significant challenge for intensivists. This study investigated the T-cell activation profile in a cohort of critically ill children with malignancies. We demonstrated that CD38 + HLA-DR + CD8+ T cells% and IFN-γ are beneficial for the early identification of children with M-HLH. In contrast, neither the macrophage activation index nor other commonly used indicators of HLH exhibit the same capacity.

Critically ill children with malignancies usually suffer from complex immune disorders induced by the primary cancer type, chemical therapy, and pathogen infection,^13^ the diagnosis of HLH depending on clinical features and traditional tests often fails to fulfill the requirement of early identification.^5^ Considering the crucial function of T-cell activation in the immune response, it is important to monitor this profile in suspected cases.^6^

CD38 + HLA-DR + CD8+ T cells%, one of the T-cell activation indexes, has been detected in various diseases related to the initiation of adaptive immunity. In viral infections such as SARS-CoV-2, dengue, Ebola, EBV, and Cytomegalovirus, both CD38 and HLA-DR were highly expressed on CD8+ T cells in patients, and the prolonged activation of CD8+ T cells may correlate with disease severity and poor outcomes.^14–17^ In 2021, Chaturvedi et al. found significantly elevated levels of CD38 + HLA-DR + CD8+ T cells in the peripheral blood and organs of children with HLH, suggesting for the first time that this index could be used as an important diagnostic parameter to differentiate HLH from early sepsis.^9^ This finding was also confirmed in a report of children with HLH caused by visceral leishmaniasis.^18^ In contrast with other types of hyperinflammatory diseases such as multisystem inflammatory syndrome in children, Kawasaki disease, Still’s disease, adenovirus, SARS-CoV-2, or parainfluenza infections, a more severe systemic inflammatory response was demonstrated in HLH or macrophage activation syndrome with a higher level of CD38 + HLA-DR + CD8+ T cells% in children.^10,19^ Compared with T cells, the macrophage activation subset (CD14 + HLA-DR+ monocytes%) exhibited markedly elevated values in both groups. The function of macrophages in malignancies is complicated because macrophages are involved in the chronic inflammatory process and also play a role in the modulation of cancer development, which may limit their application in differentiating HLH in children with malignancies.^20^

Aside from CD38 + HLA-DR + CD8+ T cells%, IFN-γ was another key index of T-cell activation in this study. Similar to our results, the value of IFN-γ in identifying pediatric HLH has also been proved in previous research.^21,22^ Interestingly, although IFN-γ is a downstream cytokine following T-cell activation, it did not always increase in parallel with CD38 + HLA-DR + CD8+ T cells%, and the cutoff value of our research was relatively low. This contradictory finding may be related to the early use of glucocorticoids, which inhibited the IFN-γ signaling pathway in activated T cells and other IFN-γ-secreting cell subpopulations.^23^

While the T-cell activation profile demonstrates potential for early detection of M-HLH, its lack of correlation with clinical severity scores warrants further exploration. Three key factors may explain this finding. First, in critically ill pediatric malignancy patients, organ failure may arise from diverse mechanisms, including tumor infiltration, chemotherapy/radiotherapy toxicity, or opportunistic infections, which could overshadow immune-mediated contributions. Second, emerging evidence suggests that coagulation abnormalities in sepsis may drive organ dysfunction beyond immune activation.^24^ Finally, immune profiling was limited to peripheral blood, which may not fully capture tissue-specific inflammatory processes in affected organs. Therefore, the immunological biomarkers must be applied judiciously when predicting outcomes in critically ill pediatric malignancy patients.

The major limitation of this study is the heterogeneity of our patients’ diseases, which resulted in a high dispersion of the data. However, regardless of the etiological factor, uncontrolled T-cell activation is a common pathway for the progression of HLH. The significance of monitoring the biomarkers in clinical practice is to provide timely evidence for early intervention. Another key limitation lies in the retrospective nature of this study. Variations in steroid regimens and incomplete follow-up data further hindered our capacity to assess dynamic changes in T-cell activation profile. Future prospective studies with larger cohorts are warranted to comprehensively characterize immune dysregulation in children with specific malignancies and to evaluate the clinical utility of T-cell activation biomarkers in guiding therapeutic strategies.

In conclusion, cytotoxic T-cell activation profile with CD38 + HLA-DR + CD8+ T cells% and IFN-γ is valuable in the early diagnosis of M-HLH in critically ill children.

## Data Availability

Due to institutional ethical restrictions and patient confidentiality agreements, the clinical datasets generated during this study are not publicly available. De-identified data may be provided by the corresponding author (email: jianzhang_scmc@126.com) upon reasonable request and approval from the ethics committee.
